# SoccerNet: A Gated Recurrent Unit-based model to predict soccer match winners

**DOI:** 10.1371/journal.pone.0288933

**Published:** 2023-08-01

**Authors:** Jassim AlMulla, Mohammad Tariqul Islam, Hamada R. H. Al-Absi, Tanvir Alam

**Affiliations:** 1 College of Science and Engineering, Hamad Bin Khalifa University, Doha, Qatar; 2 Computer Science Department, Southern Connecticut State University, New Haven, CT, United States of America; University of Wisconsin-Eau Claire, UNITED STATES

## Abstract

Winning football matches is the major goal of all football clubs in the world. Football being the most popular game in the world, many studies have been conducted to analyze and predict match winners based on players’ physical and technical performance. In this study, we analyzed the matches from the professional football league of Qatar Stars League (QSL) covering the matches held in the last ten seasons. We incorporated the highest number of professional matches from the last ten seasons covering from 2011 up to 2022 and proposed SoccerNet, a Gated Recurrent Unit (GRU)-based deep learning-based model to predict match winners with over 80% accuracy. We considered match- and player-related information captured by STATS platform in a time slot of 15 minutes. Then we analyzed players’ performance at different positions on the field at different stages of the match. Our results indicated that in QSL, the defenders’ role in matches is more dominant than midfielders and forwarders. Moreover, our analysis suggests that the last 15–30 minutes of match segments of the matches from QSL have a more significant impact on the match result than other match segments. To the best of our knowledge, the proposed model is the first DL-based model in predicting match winners from any professional football leagues in the Middle East and North Africa (MENA) region. We believe the results will support the coaching staff and team management for QSL in designing game strategies and improve the overall quality of performance of the players.

## Introduction

Football is one of the most popular sports across the globe. Advancement of tracking technology [1] has enabled us to capture every moment of professional matches, and ultimately produce a huge amount of data for analysis. The data-driven analytical approach helps the organizers to host the tournament well and the team management and coaching staff to improve their performance in upcoming games. As the hosting nation of FIFA World Cup 2022, Qatar prepared very well to organize this mega event. Recently Qatar organized a big football tournament, Amir Cup, having 20000 spectators in the stadium, successfully even during the COVID-19 pandemic maintaining all preventive measures [2]. Moreover, Qatar is emphasizing heavily on the performance improvement for the players to have a better chance of winning matches in the competition. Therefore, Qatar Football Association (QFA) is considering a data-driven approach to analyze players’ performance metrics and support the team to improve match-winning streak. Match outcome prediction based on data-driven fashion is one of the key areas of interest for every football team. Many computational methods such as statistical analysis [3], neural network-based approaches [4] and contemporary machine learning (ML) [5, 6] based approaches have already been proposed in the literature for predicting match winners from different football leagues. But there existed no literature focusing on Qatar Stars League (QSL), the only professional football league in Qatar, for match result prediction based on ML techniques. We were the first to propose the first ML model for match outcome prediction for QSL [5]. As part of this initiative, we focused on the improvement of the model. Therefore, we used a time slot-based dataset from the QSL matches for the last ten seasons and proposed a novel deep learning (DL) based method to provide a better model for predicting match outcome.

Machine learning has been used in many different fields such as facial expression recognition [7], image segmentation [8], cancer detection [9], as well as in predicting the outcome of sports including soccer. In [10], the authors used artificial neural networks to predict the outcome of basketball matches. [11] used an ensemble approach to combine the predictive abilities of Random Forest, Decision Trees, Linear Regression, Gaussian Regression, and Gradient Boosting to predict the win percentage of a team throughout a basketball game season. Going deeper with the analysis, [12] applied multi-layer perceptrons, support vector machines, and decision trees to estimate the best playing positions of players in a basketball match. Machine learning has been applied in predicting the match outcome in the game of volleyball [13, 14], baseball [15, 16], rugby [17, 18] etc., too.

In earlier works, [19, 20] used machine learning to estimate the result of soccer matches. The former used artificial neural networks trained on seven seasons of Iran Pro League (IPL) matches before applying the trained model to make inferences on matches from the 2013–14 season. The latter uses a simpler approach, a logistic regression model applied to Barclays’ Premier League season 2015/2016 to predict match outcomes and to identify variables significant in the prediction. In more recent times, the application of machine learning in soccer has become a popular topic among researchers; there have been quite a few studies on the topic. In [21], player ratings and team ratings are utilized to predict match outcomes in association football by making use of ordered logit regression (OLR) and risk modeling; while [22] takes a traditional approach by experimenting with five traditional machine learning models for predicting match outcomes in the Greek, English Premier, and Dutch football leagues. The predictive system, Dolores introduced in [23] combines two approaches—Hybrid Bayesian Networks and dynamic ratings to observe football matches in one country and predict the outcomes of matches from other countries. In [24], the authors used fuzzy-logic-based neural networks to propose a solution to the problem at hand. [25] on the other hand, used a random forest-based classifier to predict match outcomes from English Premier League. The authors in [26] used data scraped from the internet to collect match information from twelve countries and used six traditional machine learning algorithms to evaluate the performance of the proposed solution.

Within the available literature, numerous papers have been discovered that utilize machine learning (ML) models for predicting various aspects of football matches. We have specifically examined recent studies published within the last year or so, focusing on papers that delve into this particular domain. In 2019, Bilek and Ulas developed a machine learning (ML) model specifically for English Premier League matches during the 2017–2018 season [27]. Their proposed ML model, which was based on decision trees, achieved an accuracy of 67.9% when predicting match results against balanced opponents, 73.9% against stronger opponents, and 78.4% against weaker opponents. However, regardless of the opponent’s quality, the accuracy dropped to 64.8%, highlighting the importance of considering the opponent’s quality when building an ML predictor. In 2022, Malamatinos et al. employed five different ML models to predict the outcomes of football matches in the Greek League [22]. Their proposed model achieved an accuracy of approximately 67.7% in predicting match results. In 2022, Elmiligi et al. conducted an extensive study on soccer matches from 52 professional leagues over a span of 18 seasons [28]. Their proposed model achieved an accuracy of 46.6% in the test dataset. In 2023, Rico-González et al. carried out a systematic review on the application of ML models in football [29]. They focused on the use of ML algorithms in injury prediction, match winner prediction, and talent hunt prediction. The authors provided a compilation of articles that employed ML algorithms to predict match winners in various football leagues. Readers interested in the recent advancements in this field are recommended to read this article.

While there exist numerous papers that employ ML techniques to forecast football match outcomes, our primary emphasis in this paper centers around the utilization of deep learning (DL) models in the context of football. Our investigation has primarily identified two types of DL models that have been utilized for predicting football match results: LSTM (Long Short-Term Memory) and MLP (Multilayer Perceptron). LSTM is a DL model that focuses on capturing the time dependency among data points and it is widely used in modeling time series datasets. Nyquist et al. [30] used LSTM in their study published in 2017. Their study covered matches between the years 2015 and 2017 to predict “Home Win, Draw, and Away Win”. The number of matches that were included in the papers is more than 35,000. Matches from 78 competitions around the world such as English Premier League and Spanish La Liga from Europe, Major League Soccer from the USA, Serie A from Italy, and the Clubs World Cup. The study reached more than 98% accuracy in their prediction. The authors considered 15-minute interval data for the matches to build the proposed LSTM model. The LSTM model used a 50% dropout rate in the training of the model. The authors reduced the learning rate to get better results and they fixed it to 10^−4^. They tried different batch sizes between 1 and 500 for the model but decided to use a batch size of 10 for the final training. Finally, they used an embedded dimension size of 30 for the team and player’s values and kept it at 10 for the other values. It is important to emphasize that the authors used goal as a feature in their proposed LSTM-based model which supports the model to get such a high level of accuracy. Danisik et al. also considered an LSTM-based model in their work published in 2018 by [31] but the model barely reached above 52% accuracy. The authors considered football matches that were held between the years 2011 and 2016 including matches from 5 European leagues which are namely, English Premier League, French Ligue 1, German Bundesliga, Italian Serie A, and Spanish Premier Division. The LSTM model was constructed using one layer with 20% recurrent dropout, 30% forward dropout, and 32 hidden neurons. They used the sigmoid activation function and ReLU activation function on top of the LSTM layer. They fed the data in a batch size of 100 and used cross-validation for the training by adding 4 seasons and testing on another season. The proposed LSTM model considered three different classes (“win”, “lose”, and “draw”) for match result prediction. They faced an issue where draw is not predicted by the model so they tried different approaches to overcome this issue but could not achieve better results. There was no mention of the time interval used in the study for the matches, but the paper indicated that it has used previous match data as features for each match. In 2020, Rahman [32] used LSTM to predict the football match outcome using different competitions worldwide between the years 1972 to 2018. The model consisted of ten input features which are fed into the LSTM model. Then a Softmax Classifier is used to get the final results. The model did not use dropouts in the training of the model. The learning rate is around 10^−4^ which worked well for the model in the times of convergence and the time used to train the model. The batch size used in the model is between 1 and 500 but sizes above 100 were too large for the setup. The authors did not specify the time interval they considered for the LSTM model during the match. However, they indicated that they used the result of matches of the previous one to three years as features in the paper. The study has reached 63% accuracy to predict the winner of football matches using three-class match results (“win”, “lose”, “draw”). Recently, Tiwari et al. [33] considered LSTM to predict match winners in their work published in 2021. The authors included matches from the English Premier League between 2010 and 2018. The authors have tried different hyper-parameters for the LSTM model and came up with the best combination for their model which is using 10 epochs, a batch size of one, a chunk size of 27, and 512 LSTM cells in the hidden layer. The authors considered two-class classification (“win”, “loss”) but it is important to emphasize that authors have used goal as one of the features in their model. The proposed method achieved 80.75% accuracy in the prediction.

**Fig 1:**
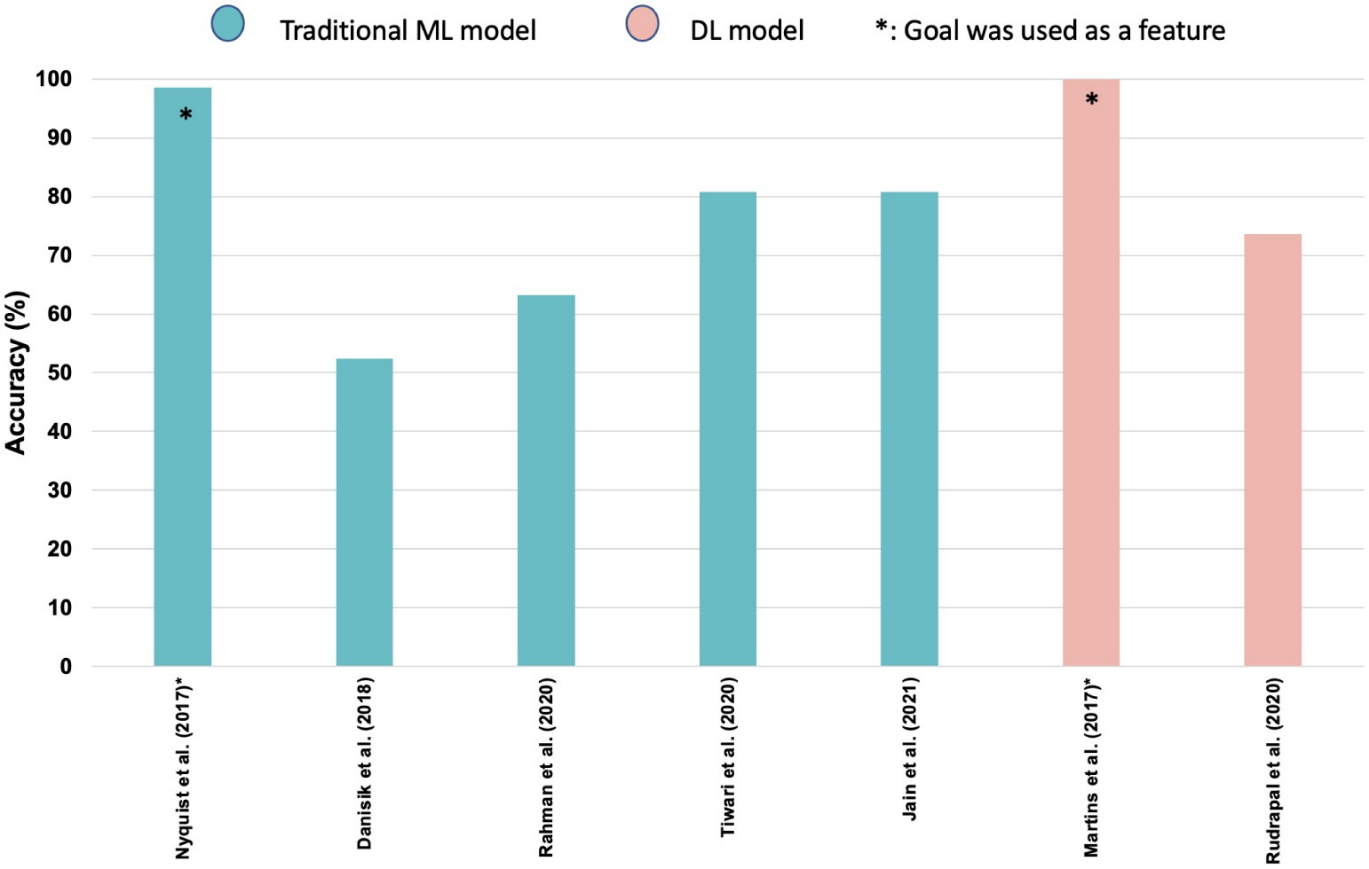
Performance comparison of the existing machine learning-based models for soccer match outcome prediction.

Apart from the LSTM-based model, we found few other papers that investigated MLP as the model to predict the outcome of football matches. Martins et al. [34] used MLP among other models to predict the winner of football matches in some leagues around the world. They used 10 hidden stats in the training set which 70% of the dataset and 30% is the test set. The study covered matches from 2010 to 2015 and has reached an accuracy of 100% for MLP for some leagues. It is important to emphasize that the authors used goal as one of the features in the match result prediction as like in [30]. Rudrapal et al. [35] have also used MLP as a model to predict the outcome of football matches in the English Premier League. The authors considered three groups of features namely, team-related, player-related features, and head-to-head match-related features to build a machine-learning model for predicting match winners. The authors applied four different ML models namely, MLP, SVM, Gaussian Naive Bayes, and Random Forest for this purpose. The study covered matches from 2000 to 2016, and the proposed final model based on MLP reached an accuracy of 73.6%. Fig 1 summarizes the performance of deep learning-based models proposed for the match result prediction in literature.

From Table 1, we can observe that there exists evidence in the literature for match result predictors from different professional football leagues. But there exists no literature which proposed DL-based models for the same purpose. Therefore, we proposed a DL-based method to predict match winners for football matches in QSL. Hence, our primary motivation for this work was to utilize the power of modern machine learning—deep learning to produce state-of-the-art results in predicting the outcome of a soccer match in a professional league. In addition, we also wanted to present additional insight into the problem by analyzing both the individual and the joint impact of different playing positions and match segments on the outcome. Moreover, another motivation for the work was to use accumulative match segments to predict the outcome to see which part of the match played the most crucial role in the final scoreline.

**Table 1:** Accuracy of predicting football match outcome using deep-learning based models.

Ref.	Competition(s)	Years	Total matches	Feature count	Temporal aspect	DL Model	Accuracy
[30]	Included 78 competitions from around the world such as English Premier League, Spanish La Liga, Serie A from Brazil, and Clubs World Cup.	2015–2017	35,000	NA	15 Min interval within a match	LSTM	98.6%*
[31]	Five different leagues in Europe (English Premier League, French Ligue 1,German Bundesliga, Italian Seria A,and Spanish Premiera Division)	2011–2016	NA	134	Focusing on previous match’s result as input for current match	LSTM	52.40%
[32]	Leagues and competitions from around the world	1872–2018	NA	NA	Using current result compared to previous years results (1–3 years)	LSTM	63.30%
[33]	English Premier League	2010–2018	NA	60	NA	LSTM	80.80%
[34]	English Premier League, La Liga, Brazilian League Championships	2010–2015	1520	54	NA	MLP	100%*
[35]	English Premier League	2000–2016	11,400	40	NA	MLP	73.60%
**SoccerNet**	Qatar Stars League	2012–2022	1,462	22	15-minute match segments	GRU	82.30%

*: Authors used “Goal” as a feature to predict the winner of the match; NA: Not available in the literature;

The contribution of the is work can be summarized as follows:

1. We have incorporated the highest number of seasons from QSL to cover the most number of matches as part of our analysis. We, for the very first time, have used a time slot of 15-minute intervals based data from QSL and analyze players’ performance in different stages of the match.
2. We propose a DL-based model which considers players’ performance in different time slots of the game to predict match outcomes. The proposed model provides the best result in predicting match outcomes from QSL. To the best of our knowledge, the proposed model is the first DL-based model in predicting match winners from any professional football leagues in the Middle East and North Africa (MENA) region.
3. We showed that in QSL, defenders play a major role in winning matches. The contribution of defenders is more dominant than midfielders and forwarders. Moreover, our analysis suggests that the last 15 to 30-minute segment of the match has a more significant impact on the results than other match segments.

The rest of the paper is organized as follows. We discuss the statement on ethical approval, dataset collection and preprocessing, a detailed description of the experiment configuration and the candidate models, as well as the proposed model (SoccerNet), and the evaluation metrics in the Materials and Methods section. A statistical analysis of the dataset, the outcome of our experiment as well a detailed ablation study are presented in the Results section. The Discussion section contains analysis of the results, a comparison with other applicable methods, and the limitations of our proposed approach. The Conclusion and the remaining sections are the closure to this research article.

## Materials and methods

### Ethical approval

This study was designed and conducted under the policy and regulation of the Ministry of Public Health (MoPH), Qatar. All the ethical aspects were approved by the Institutional Review Board (IRB) of Qatar Biomedical Research Institute (QBRI), under Hamad Bin Khalifa University (HBKU), Qatar. In this retrospective study, we used only de-identified information of players’ match time performance with consent from QSL and QFA for the dataset.

### Dataset collection

We used a dataset for the professional football matches of the Qatar Stars League (QSL). QSL is the top-ranked league in Qatar, which usually involves over fourteen teams per season. During the match time, players’ performance metrics and other metrics were captured based on the Stats Perform platform [36] which is already set up in multiple stadiums in Qatar. The dataset covered the matches of QSL from the last ten seasons, starting from 2012 up to 2022. In the dataset, we found in total of 19 football teams who participated in QSL during this period. The basic statistics about the dataset containing season, the number of matches, number of teams participated in QSL are highlighted in Table 2.

**Table 2:** Summary statistics about the dataset.

Season	Total Matches	Total Teams
2012–13	78	12
2013–14	124	14
2014–15	134	14
2015–16	130	14
2016–17	135	14
2017–18	105	12
2018–19	108	12
2019–20	88	12
2020–21	106	12
2021–22	94	12

For each match, STATS platform captured the summary of players’ performance metrics over a 15-minute time interval. Hence, during a 90-minute standard match, the platform captured the summary data for the 0–15 mins (t1), 16–30 mins (t2), 31–45 mins (t3), 46–60 mins (t4), 61–75 mins (t5) and 76–90 mins (t6). The system also captures data for the extra time—(45+ minutes at the end of the first half and 90+ min at the end of the second half). For knockout matches, it also captures data in the same fashion at 15 mins intervals after 90 mins. In our analysis, we consider all data that are captured during 0–90 mins with 15 mins time intervals. In the dataset, we had the data from each player and related match information. For each match, we had match-related information such as match date, venue, season, teams, and scoreline. For each 15 mins time interval, we had both the players’ technical and physical performance metrics. A summary of the information from the dataset is shown in Table 3.

**Table 3:** High-level summary of the information (feature) available in the dataset.

Information	Brief summary
**Match related**	**Summary of full match time**
Season	It contains the data from the season 2012–13 upto 2021–22
Scoreline	Score from both team
Venue	Stadium in Qatar
Playing teams	Name of the playing teams
**Players’ technical performance (16)**	**15 mins time interval information**
Foul	Number of fouls committed by a player.
Tackle	Tackles by players to dispose of opponents.
Clearance	Moving away the ball from the current area.
Yellow card	Number of yellow cards received by a player.
Red card	Number of yellow cards received by a player.
Offside	Number of times a player faced offside during a match.
Shots on target	Number of shots made by a player on target.
Shots missed target	Number of shots by a player that missed the target.
Successful pass	Number of passes that reach teammates.
Unsuccessful pass	Number of passes that could not reach teammates.
Corner	Number of corners made by a player.
Cross	A pass aimed at a teammate to reach in front of the opponent’s goal.
Dribble	Player maneuvers the ball around the defender of the opposing team.
Free kick	Number of free kicks made by a player.
Interception	Number of times a player steals the ball from an opponent.
Number of players at each position	Number of player playing in each position, defense, midfield, and forward
**Players’ physical performance (6)**	**15 mins time interval information**
Standing distance	Speed in the range of 0–1 km/hr to cover the distance.
Low-speed distance	Speed in the range of 1–6 km/hr to cover the distance.
Moderate-speed distance	Speed in the range of 6–15 km/hr to cover the distance.
Elevated-speed distance	Speed in the range of 15–20 km/hr to cover the distance.
High-speed distance	Speed in the range of 20–25 km/hr to cover the distance.
Very high-speed distance	Speed above 25 km/hr to cover the distance.

### Experiment setup

To extensively evaluate our proposed method against other approaches, we modeled the problem in two ways. In the first approach, we considered the data as tabular, devoid of any temporal aspect. In the second approach, we treated the data as a sequence of feature samples taken at different time intervals. For each of these two approaches, we trained, evaluated, and tested multiple machine-learning models to find the best one.

#### Tabular feature based approach

In the tabular-based approach, we assumed that the dataset is not temporal in nature. That is, each data point xi is an unordered collection of features spanning the six match segments: 0–15, 16–30, 31–45, 46–60, 61–75, and 76–90 minutes from a soccer game. Since there are 22 features (16 features from players’ technical performance and 6 features from players’ physical performance, see Table 3), we used these 22 features for each of the 6-time intervals, resulting in each data point of size of a 132 (=22x6) dimensional vector for each player position. When combined into a single feature vector representing data from all (defender, midfielder, and forward) player positions, we get a 396 (22 x 6 x 3) dimensional vector. For any match, if all features from any position were missing, we dropped that match. For the remaining features, we replaced the missing values by considering the median value of the corresponding feature. For the 949 matches in the dataset, we obtained an 1898 x396 data matrix where the first dimension corresponds to match records (equal to twice the number of matches since each match contributes twice—once for each team) and the second to features.

#### Sequence-based approach

In the sequence-based approach, we assumed that the dataset does exhibit temporal properties. This is inherent in the nature of the problem as a soccer match is a sequential event. We arranged the data points to reflect this by separating match information over the match segments. That is, we treated the set of feature values from each match segment as a time-step input to our sequence model. Based on whether the information from each player position was analyzed separately or together in each time step, we can further segregate the type of experiment we performed into the following two categories:

1. **Position-agnostic sequence model:**
  In the position-agnostic model, we combined the data from all player positions to be processed together in each time step. Hence, if *x*_*t*_ denotes the feature vector processed at time step t, then *x*_*t*_ is a 3*22 element vector with the feature values from the three positions—defender, midfielder, and forward concatenated together. Our motivation behind experimenting with the position-agnostic model was to find out if a unified model that takes information from multiple positions together would perform better than one that does not, which is explained next.
2. **Position-aware sequence model:**
  The position-aware model treats data from each player’s position as a separate piece of information. The models in this category use a three-pronged pipeline that takes data from all three player positions as input but segregates them into three parts according to the player position. Each part then follows a similar, but non-identical modeling pipeline before merging into a common head. If we represent the feature values being processed at time t for position p with ^*p*^*x*_*t*_, then ^*p*^*x*_*t*_ is a 22-element vector representing the feature values from only position p. A model implementing this pipeline is position-aware since data from each position is processed separately.

### Candidate model description

In this section, we describe the models that were used to predict soccer match outcomes. We considered primarily three types of models as candidates: (1) traditional feature-based machine learning models, (2) position-agnostic neural network-based models, and (3) position-aware neural network-based models. Each of them is described below.

1. **Tabular Feature-based machine learning models:**
  For tabular feature-based models, we used 396- (22 feature x 6 math segment x 3 playing positions) dimensional vectors as the input. We normalized the features based on min-max normalization using statistics from the training set. We experimented with four traditional (non-deep neural networks) models, namely decision trees (DT), random forest (RF), XGBoost (XGB), and CatBoost (CatB) models for predicting match winners. The reason we did not include a multi-layer perceptron in our analyses is due to ensemble, especially gradient boosting-based algorithms that can still surpass MLP in tabular data analysis [14]. All the hyperparameters of the models were tuned using Random Search from the Scikit-Learn package in Python. S1 Table highlights the list of hyperparameters for the tabular feature-based baseline models.
2. **Position-agnostic neural network-based models:**
  The position-agnostic sequence models we experimented with are all based on deep neural networks. We used the popular sequence-based neural networks such as the LSTM, GRU as stand-alone networks as well as in a combined setting with convolutional layers. The four resulting network descriptions are given below along with the hyperparameter configurations.
  - **GRUNet:** This GRU-based network we used has a four-layer GRU in the first processing layer followed by two linear (fully-connected) layers. Each GRU layer having a 600-dimensional hidden vector passes between one time step to the next to carry the information represented thus far. The fully-connected layers have 4*600 and 128 neurons, respectively. We used batch normalization, dropout (p = 0.6), and the ReLU nonlinearity after each layer except for the last, which has only a sigmoid activation layer.
  - **LSTMNet:** Our unified LSTM-based candidate is identical to GRUNet, except for the GRU module replacement with an LSTM module with the same number of layers.
  - **CNN-GRUNet:** While the previous two candidate models apply sequential processing directly to the input data, GRU the CNN-based sequence agnostic models first pass the input through a convolutional layer before the sequence processing steps as an additional processing stage. This results in a network that starts with a convolutional layer with a single, channel-spanning filter that allows for a non-linear mixing of the player-position data without altering the number of features (22). It is followed by a 4-layer GRU with a 600-dimensional hidden state before finally ending in two fully-connected layers of size 4*600 and 128, respectively. We followed the standard procedure of adding batch normalization, dropout (after linear layers only), and ReLU activations after all but the final layer in the network.
  - **CNN-LSTMNet:** The final candidate model in the position agnostic category is a simple modification of CNNGRUNet where an LSTM module replaces a GRU module. The rest of the configurations are identical.
3. **Position-aware neural network-based models:**
  The position-aware sequence models consider three playing positions as separate data streams for building deep learning models. In this experiment setup, we considered both LSTM and GRU-based networks, and ultimately, the GRU-based position-aware network (we named it as SoccerNet) became our proposed model in the present work. In the next section, we explain it in more detail.

### Proposed position-aware model SoccerNet

**Fig 2:**
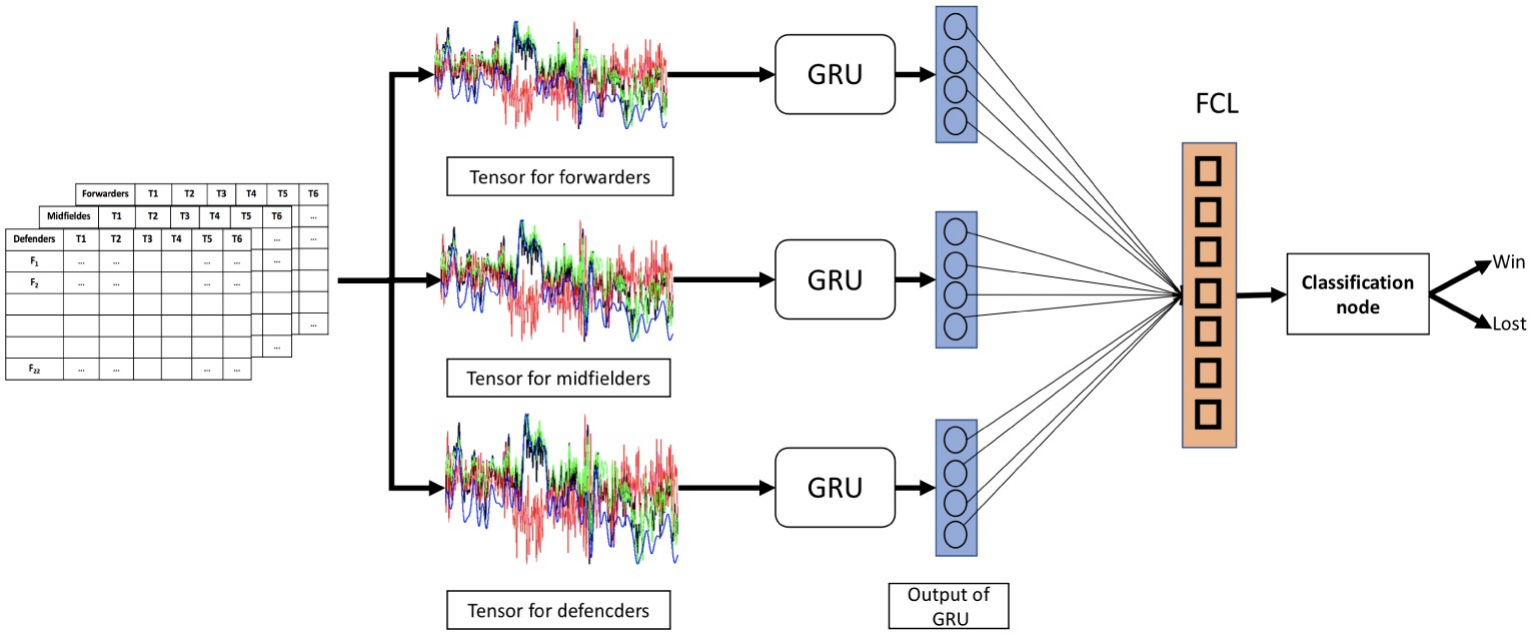
Proposed architecture for the SoccerNet for predicting match winners. FCL: Fully connected layer. GRU: Gated Recurrent Unit.

In the proposed position-aware model, we considered each playing position, i.e., defend, midfield, and forward as separate data streams. For each of the data streams, we generated tensors and passed them through sequence-based networks. For sequence-based networks, we used both LSTM- and GRU-based models. Among them, GRU-based network provided better accuracy in match result predictions (see Results section). Therefore, GRU based model was proposed as the final SoccerNet in this present article. The proposed SoccerNet network architecture is shown in Fig 2.

The input layer is a four-dimensional data structure (3, #samples, 6, 22) storing the twenty-two input features from three player positions (defender, midfielder, forward) across six time slots, each spanning fifteen minutes from the match. In the first processing layer of the network, input data from each player position passes through two-layer GRUs with 600 hidden states each. The outputs from the last time step of the GRUs are then concatenated into a vector that stores an intermediate representation of the match computed from the three player positions. This concatenated vector is then passed through a linear layer, a batch normalization layer, and a ReLU activation layer. The output from this activation layer then finally passes through a final linear layer followed by a sigmoid layer to produce the output of the network. The output a indicates the probability *P*(*Win*|*w*, *d*); that the team with match statistics d won the match (here w represents the parameters of the network and d the input to the network).

**Fig 3:**
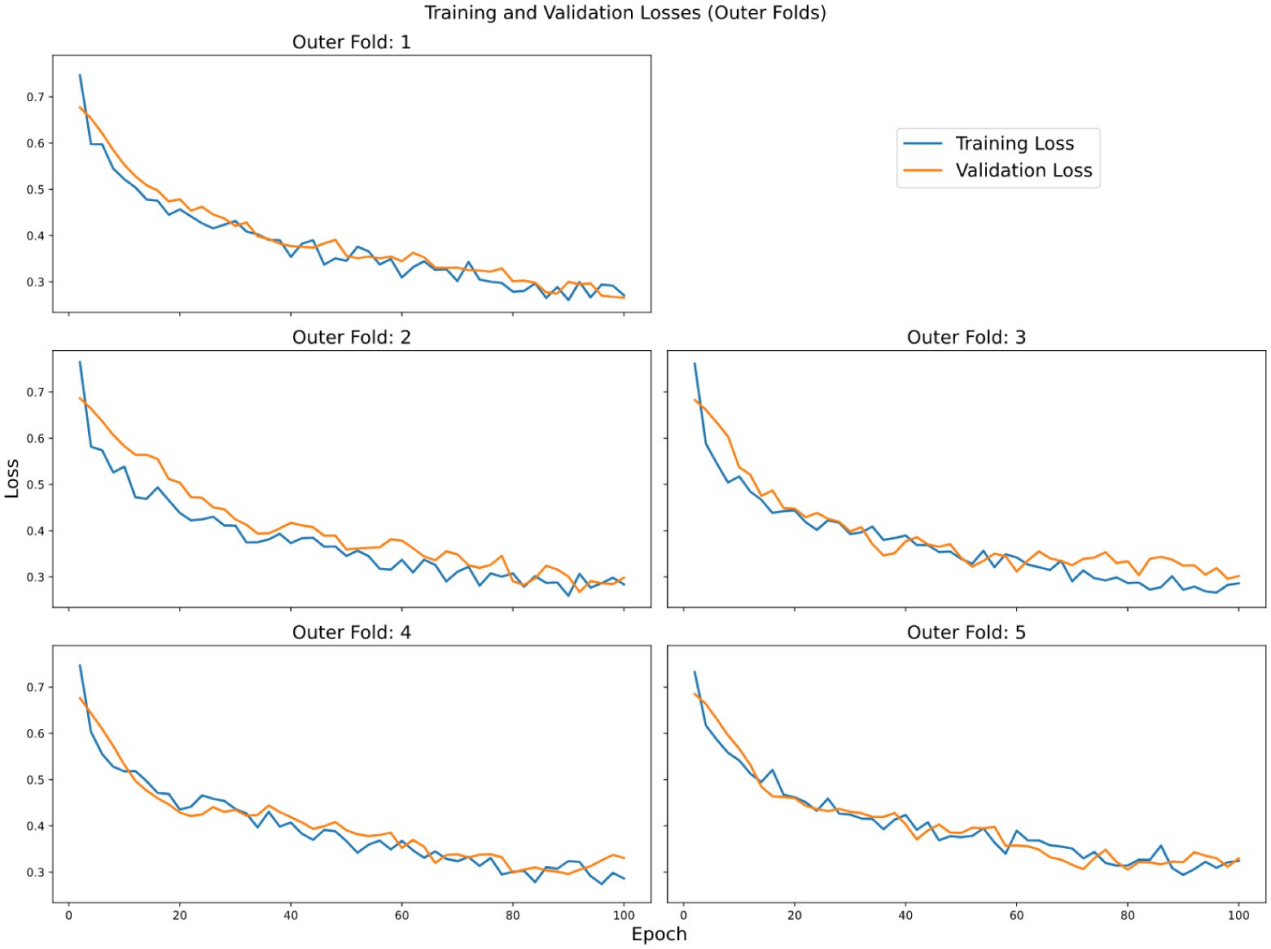
The training and validation losses from the outer folds of the experiment.

The network was optimized for 100 epochs using the Adam optimization algorithm [37] while the learning rate was modified using the One Cycle Learning Rate Scheduler [38]. We extensively experimented with the hyperparameters (e.g., the number of layers in GRUs, hidden state size, and number of neurons in the linear layers) and settled with the ones mentioned earlier. We used mini-batch gradient descent for parameter updates with a batch size of 64. To make the findings statistically stable, we applied nested cross-validation [39] with 5 folds in both the inner and outer configuration. On a machine with an AMD Ryzen 7 5800X, 64 GB of DDR4 dual-channel memory, and an NVIDIA RTX 3090 GPU, for the final model, each experiment takes 4 hours to complete. The total number of parameters in our proposed model is 103,27,242. Parameter counts for each layer of the network are shown in Table 4. The training and validation losses can be seen in Fig 3.

**Table 4:** Number of parameters in each layer of our proposed model.

Layer	Parameter Count
GRU (Forwarder)	32,88,600
GRU (Midfielder)	32,88,600
GRU (Defender)	32,88,600
Fully-Connected Layer 1	460,928
Batch Normalization Layer	256
Fully-Connected Layer 2	258

### Model evaluation

For the machine learning (ML) based model, we used 10-fold cross-validation (CV) to train, validate and test the model. In summary for each fold, we used 80% of the data sample for training, 10% for validation and the remaining 10% as a test set. We considered the following metrics for evaluating the performance of the models:

$$Accuracy=\frac{TP+TN}{TP+FP+TN+FN}$$ (1)

$$Precision=\frac{TP}{TP+FP}$$ (2)

$$Sensitivity=\frac{TP}{TP+FN}$$ (3)

$$Specificity=\frac{TN}{FP+TN}$$ (4)

$$F1-score=\frac{2*Precision*Recall}{Precision+Recall}$$ (5)

$$MCC=\frac{\left(TP*TN)-(FP*FN\right)}{\sqrt{\left(TP+FP)(FP+FN)(TN+FP)(TN+FN\right)}}$$ (6)

Where TP, TN, FP, FN stands for the number of true positive, true negative, false positive, and false negative samples, respectively.

## Results

### Performance comparison for the tabular-feature-based, position-agnostic and position-aware models

In Table 5 below, we show the results from the candidate models and the proposed model. For each model, we computed the following evaluation metrics: accuracy, precision, sensitivity, specificity, F1 score, Matthew’s Correlation Coefficient (MCC), and Area Under the Curve for the Receiver-Operator Characteristics curve (AUC-ROC).

**Table 5:** Performance of ML models on test set for both tabular and sequential (position-agnostic and position-aware) approaches on test set.

Model	Accuracy	Precision	Sensitivity	Specificity	F1_Score	MCC	AUC_ROC
CatBoost	65.15±0.72	60.42±0.58	92.99±0.50	37.31±1.79	72.96±0.35	36.32±1.24	65.15±0.73
DT	59.51±0.20	59.28±0.22	60.93±0.19	58.09±0.31	60.08±0.18	19.04±0.40	59.51±0.20
RF	72.02±0.20	71.23±0.27	74.16±0.37	69.89±0.47	72.6±0.20	44.18±0.40	72.03±0.20
XGBoost	76.04±0.08	75.71±0.06	76.7±0.24	75.38±0.13	76.19±0.11	52.1±0.16	76.04±0.08
**Position-agnostic**							
GRUNet	79.24±0.26	80.08±0.06	77.92±0.51	80.56±0.13	78.9±0.31	58.6±0.51	79.24±0.26
LSTMNet	77.31±0.26	77.27±0.29	77.61±0.55	76.99±0.43	77.32±0.29	54.8±0.52	77.3±0.24
CNN—GRUNet	78.64±0.26	77.84±0.46	80.87±0.23	76.38±0.77	79.1±0.22	57.69±0.47	78.62±0.27
CNN—LSTMNet	76.09±0.59	75.9±0.61	76.69±0.74	75.49±0.68	76.17±0.61	52.35±1.18	76.09±0.59
**Position-specific**							
SoccerNet (LSTM)	79.7±0.28	79.33±0.22	80.36±0.53	79.04±0.28	79.77±0.33	59.51±0.55	79.7±0.28
SoccerNet (GRU)	80.77±0.22	80.34±0.13	81.68±0.39	79.85±0.41	80.93±0.22	61.67±0.44	80.77±0.22

Among the tabular models that ignored the time-sequence aspect of the data, the XGBoost [40] implementation of the Gradient-Boosted Model performed the best in all evaluation metrics. It reached an accuracy of 76.04%, which is more than 4% from the next best model Random Forest. XGBoost beats Random Forest in other evaluation metrics by at least 2.5%, on average by more than 4%.

For the position-agnostic models, which combined all three positions into a single input through GRUNet and LSTMNet achieved the best performance in the category with 79.24%, and 77.31% accuracy, respectively. On the other hand, when a convolutional layer was added before the GRU or LSTM layer, both models CNN-GRUNet and CNN-LSTMNet showed relatively lower accuracy (78.64% and 76.09%) compared to their counterparts, GRUNet and LSTMNet, respectively.

For the position-aware models, we considered two networks: a GRU-based (SoccerNet—our proposed model) network and an LSTM-variant of it. SoccerNet performs the best among all models in the experiment with an accuracy of 80.77%, while its LSTM-counterpart scored 79.7% accuracy. SoccerNet beat all models in all evaluation metrics except for sensitivity, where CatBoost was the top scorer. However, in doing so, CatBoost had to significantly sacrifice the prediction performance in the negative class (Loss) which resulted in a specificity of only 37.31%, whereas SoccerNet reached 79.85% in the same metric. Hence, SoccerNet is a more robust model in all aspects.

### Ablation study

In this section, we discuss the results from different perspectives by performing ablation studies. Specifically, we are interested in (i) exploring the effect of a player’s position in predicting the match outcome. (ii) understanding how different segments from the match determine the outcome, and (iii) the joint effect of position and match segments on the match outcome. Each of these three approaches is discussed in detail in the following subsections.

#### Ablation study on players’ position

Our proposed model GRUNet uses information from three player positions: defender, midfielder, and forward. Hence, it is a holistic approach in terms of player position. We think it will be worthwhile to investigate to what extent player positions affect the outcome of the match. To this end, we performed an ablation study of GRUNet on the player positions. We experimented with six configurations, each considering either a single position or a pair of positions. The results are shown in Table 6. We will first discuss the performance of the single-position models, then the pair-position models.

**Table 6:** The effect of different player positions on the outcome.

Position	Accuracy	Precision	Sensitivity	Specificity	F1_Score	MCC	AUC_ROC
Defender	76.35±0.25	75.54±0.29	78.03±0.28	74.67±0.38	76.73±0.23	52.77±0.50	76.35±0.25
Midfielder	67.50±0.15	67.47±0.23	67.85±0.31	67.15±0.43	67.59±0.14	35.07±0.29	67.50±0.15
Forward	67.75±0.31	68.33±0.32	66.33±0.48	69.18±0.40	67.25±0.35	35.56±0.61	67.75±0.31
Defender + Midfielder	77.36±0.30	76.94±0.33	78.23±0.41	76.50±0.44	77.53±0.31	54.78±0.60	77.36±0.30
Midfielder + Forwarder	72.94±0.24	73.99±0.26	71.11±0.63	74.76±0.46	72.33±0.34	46.11±0.47	72.94±0.24
Defender + Forwarder	78.07±0.24	77.67±0.21	78.83±0.40	77.31±0.26	78.21±0.26	56.20±0.47	78.07±0.24
All three positions	80.77±0.22	80.34+ 0.13	81.68±0.39	79.85±0.41	80.93+ 0.22	61.67±0.44	80.77±0.22

We can see that the single-position model performs best in the defender position. Hence, the defenders influence the results of a match the most. This might seem contrary to our natural tendency to think that most of the time, the forwards (and often midfielders) score goals in a soccer match and consequently, have a more significant impact on the outcome of a soccer match compared to their defender teammates. However, the dataset we used in this work actually supports this alternative finding as matches played in QSL are more defense-oriented [5] than, for example, the English Premier League and Spanish La Liga where aggressive gameplay is mainly contributed by the forwarders [41]. The results also demonstrate that midfielders and forwards have a comparable impact on the result of a match.

It is also evident from the results that pair-position models perform better when the defender position is included in the analysis. This is consistent with the findings from the single-position model. However, the difference in performance between the models that include the defender position and the one that does not is lower (4% to 5%) in pair-position models than the same in single-position models (8.5%), which tells us that match outcome prediction benefits from including forward (78%) and midfielder data (77%) (Table 6). Our ablation study in this section is consistent with the reasoning that match outcome prediction is more accurate (refer to Table 5) when more player positions are considered.

#### Ablation study on the selected match segment

The experiments shown so far consider information from the entire match to predict the match outcome. Hence, the sequence analysis module in our proposed architecture sees all 15-minute match segments (1 to 6, inclusive). We now shift our focus to understanding how the player information from different segments of the match is related to the match results. We approach this task by designing experiments that consider different numbers of timeslots, starting both from the beginning of the match and the end of the match. This results in ten configurations in this partial match analysis experiment with the following time lengths: 15 minutes, 30 minutes, 45 minutes, 60 minutes, 75 minutes, and 90 minutes, each from the beginning and the end of the match as mentioned earlier. The results are shown in Table 7.

**Table 7:** The effect of different match segments on the outcome considering test set using the final model (based on GRU Net).

Match segment	Accuracy	Precision	Sensitivity	Specificity	F1_Score	MCC	AUC_ROC
First 15 mins	64.49±0.36	64.77±0.42	64.29±0.50	64.70±0.69	64.40±0.34	29.09±0.72	64.49±0.36
First 30 mins	64.90±0.33	64.67±0.33	65.83±0.45	63.99±0.41	65.20±0.34	29.87±0.66	64.91±0.33
First 45 mins	69.43±0.34	69.14±0.39	70.61±0.42	68.26±0.56	69.79±0.32	38.96±0.69	69.43±0.34
First 60 mins	68.97±0.17	69.44±0.30	68.37±0.49	69.58±0.55	68.73±0.22	38.12±0.34	68.98±0.17
First 75 mins	73.80±0.23	73.54±0.26	74.57±0.43	73.04±0.41	73.97±0.24	47.72±0.46	73.81±0.23
Full time	80.77±0.22	80.34±0.29	81.68±0.39	79.85±0.41	80.93±0.22	61.67±0.44	80.77±0.22
Last 75 mins	77.21±0.33	76.99±0.35	77.82±0.50	76.60±0.47	77.32±0.34	54.55±0.65	77.21±0.33
Last 60 mins	74.31±0.32	74.48±0.41	74.46±0.52	74.16±0.62	74.32±0.32	48.81±0.63	74.31±0.32
Last 45 mins	72.58±0.24	72.30±0.26	73.34±0.42	71.82±0.37	72.76±0.27	45.24±0.48	72.58±0.24
Last 30 mins	72.17±0.27	71.82±0.27	73.15±0.50	71.21±0.41	72.39±0.31	44.46±0.54	72.18±0.27
Last 15 mins	69.69±0.20	69.08±0.22	71.42±0.36	67.96±0.35	70.18±0.22	39.47±0.40	69.69±0.20

If we initially limit ourselves to analyzing results from configurations that consider a decreasing number of timeslots with truncations taking place from the beginning of the match, we can see that the last 30-minutes of the match and the last 15-minutes of the first half are more informative than the other time slots since the performance decreases by 7%, 5%, and 5% when we leave out the last 15-minutes, 60 to 75 minutes, and 30–45 minutes, respectively, whereas the performance drop by leaving the other time slots never exceed 1.6%. This is consistent with how the game is played; for example, players try to gain an upper hand against the opposition in the last 15 minutes of the first half to go into the interval with a psychological advantage. The performance of the players in the final 30 minutes also often seals the outcome since a goal scored in this part of the game is hard to come back from by the opposition.

As our analysis shows, the last 15-minute segment of the match has a significant impact on the results as leaving out information from this segment causes the performance to decrease by more than 7% from the model accuracy that uses the full-match data. Comparatively, the first 15 minutes have a lower impact as the corresponding drop in performance is only around 3.5%. Similarly, the 60–75 minute segment (the second-to-last 15-minute timeslot) of the match is more important for the results than the 16–30 minute segment (the second 15-minute timeslot) as evidenced by the corresponding performance drop of 4.8% versus 2.9%. This makes sense since often the determining actions take place in the second half of the match.

#### Ablation study on players’ position on different match segment

Now that we have investigated the individual effects of different player position settings and match segments on the prediction performance. We aim to gain additional insights about the performance conditioned on the joint distribution of players’ positions and match segments. To this end, we trained, validated, and tested our proposed model on configurations from the grid match segments x player positions. Since there are 11 match segments (including the full length of the match) and 6 player position settings (3 individual and 3 pairwise), this results in 66 experiments in total. The results are shown in S2 Table.

**Fig 4:**
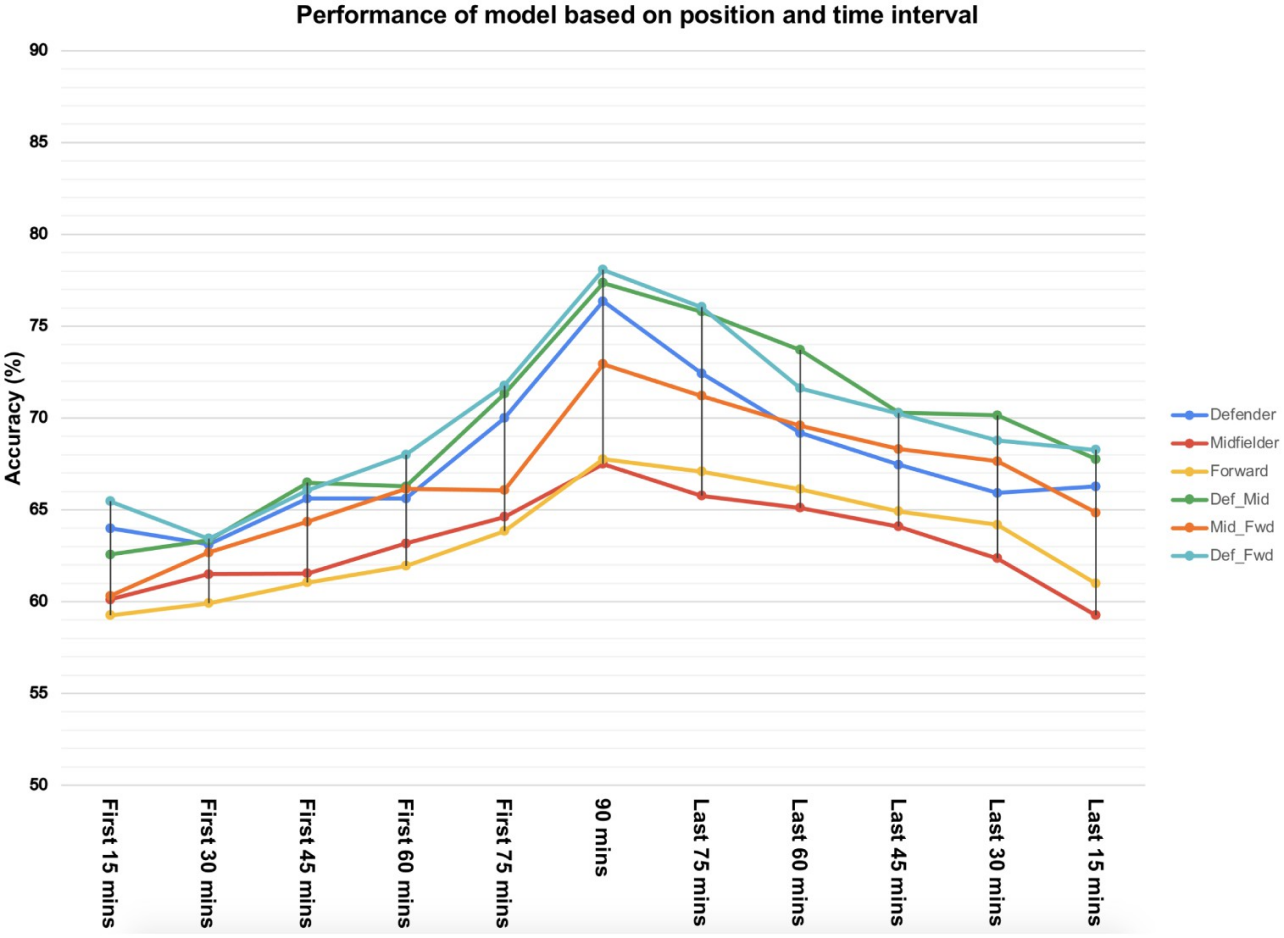
Performance of the proposed model considering players’ position and match segment.

Fig 4 shows the model performances on different player positions and match segments. The left halves of the plots in general lie below the corresponding right halves, which indicates that the performance of the first n minutes has a lower impact than the last (90-n) minutes. This is consistent with real-world observation where matches get more intense as the full-time approaches. Fig 4 supports the same observation we noted in Table 3 where the performance of the model improves when defenders are included in the analysis irrespective of the match segment considered. Whenever the defender position is involved (in both single- and pair-position cases), the model performance is better than the counterpart. Moreover, defender-based models can predict the outcome more accurately even from the first n minutes of the match than the last (90-n) minutes when only the midfielder or forwarder position is considered. This is evident from Fig 4 as the left halves of lines corresponding to configurations involving a defender largely lie above the right halves of the lines corresponding to the configurations that do not.

## Discussions

### Principal findings

In our study, we considered the highest number of seasons covering from 2021—2022 to analyze more than 949 football matches to predict match winners from QSL professional matches. To the best of our knowledge, this study covers the largest number of seasons to cover any professional football league in the MENA region. The proposed GRU-based model, SoccerNet, was able to predict match winners at over 82% accuracy. This indicates the superiority of the proposed DL-based outperforming the existing DL-based models for predicting soccer match results from other professional leagues. Moreover, we showed that when we build the model considering the players’ position (position-aware model), it shows better accuracy compared to the models which are position-agnostic, emphasizing the importance of players’ role from different positions (Table 5). We also build four different traditional feature-based machine learning models DT, RF, XGBoost, CatBoost for predicting match results (Table 5). But the traditional machine learning-based models were not able to outperform the proposed GRU-based SoccerNet model. This emphasizes the importance of incorporating DL models for predicting soccer match results.

Considering the ablation study on different playing positions, we observed that defenders play a more significant role in winning matches than midfielders and forwarders at QSL matches (Table 6). Moreover, the joint contribution of defenders with midfielders or forwarders played a crucial role in winning matches compared to the joint contribution of midfielders and forwarders.

Considering the ablation study on different match segments, we highlighted that the last 15-minute match segment had the most significant impact on the determination of match results. On the other hand, the first 15-minute match segment had the least impact on match results (Table 7). But considering the full match time with all match segments provided the best accuracy for SoccerNet (Table 7).

Moreover, when we combine playing positions with different match segments, we observe the role of defenders dominating over midfielders and forwarders in all match segments (Fig 4). This clearly indicates the QSL match results are highly dominated by the performance of defenders in all match segments. In summary, our results summarize the contribution of playing positions, match segment, and the combination of them considering the largest number of professional football matches from QSL.

### Comparison against other methods

We investigated the existence of previous studies that focused on QSL matches for match-winner prediction. Based on our literature search, we only found our previous study, where we developed a machine learning model for the same purpose on matches from QSL for seven seasons and it achieved nearly 80% accuracy [5]. Our previous study covered seven seasons from QSL to predict match winners based on logistic regression (LR) based models. But the dataset, considered in our previous study, was missing match segment-wise players’ performance metrics. Therefore, the current result is not systematically comparable to the results from our previous article. But we considered a similar approach by considering the tabular feature-based model where the XGB model achieved the best performance with over 76% accuracy (Table 5). Therefore, the proposed DL-based method in the present article achieved the best result for predicting soccer match results with the highest accuracy for QSL matches. Moreover, we included the performance of the DL-based soccer match result predictors from the literature in Table 1. We can observe that multiple studies proposed DL-based models for predicting soccer match results for English Premier League, La Liga, and Brazilian League. But none of the DL-based methods focused on QSL. Moreover, the overall performance of the previously proposed DL-based model achieved nearly 80% accuracy except for a few cases where the authors considered “Goal” as a feature of the proposed model. So, our proposed DL-based model achieved the state-of-the-art result for QSL match result prediction with over 82% accuracy.

### Limitations

Our study considered professional football matches that were held in Qatar under QSL. So, results are specific to QSL only and might not be generalized for other professional leagues. As we do not have access to datasets from other professional leagues, we could not verify it for those leagues. Moreover, Qatar being a small country, the concept of home or away game is not applicable here. The majority of the teams have their own stadium, but the games are arranged in neutral venues as well. Overall, considering the close vicinity of stadiums, the effect of home or away games is minimal.

## Conclusion

In the present article, we proposed a DL-based method, SoccerNet, to predict the football match results in QSL considering players’ performance metrics. We showed that DL-based models provide a better result in predicting match results compared to traditional feature-based machine learning models. The proposed model achieved over 82% accuracy in predicting the matching winner for QSL football matches. The proposed model also suggested the importance of different match segments and highlighted the relative importance of the later part of the match to decide results. We also demonstrated that players in different field positions have different levels of contribution to match results. We also showed that defenders play a significant role in winning matches at QSL. Overall, we believe our results will support the coaching staff and team management to improve the players’ match performance and support to focus on the key area for their improvement.

In the future, based on the availability of data from QSL, we plan to cover more seasons to include an increased number of matches. Moreover, we will investigate other deep learning-based models that are performing well such as transformer-based networks, attention mechanisms applied to LSTMs/GRUs, etc. which could improve the performance of the proposed model. In our present work, we could not consider the drawn matches as part of our analysis as it was not part of the dataset. In the future, we will incorporate drawn matches and develop a solution for match outcome prediction modeling it as a multi-class classification problem.

## Supporting information

S1 Table List of hyperparameters for the tabular feature-based baseline machine learning models. (XLSX)

S2 Table Match outcome prediction using different player position configurations and match segments (The joint effect of different match segments and player positions on the outcome). (XLSX)

## References

[1] Almulla J, Takiddin A, Househ M. The use of technology in tracking soccer players’ health performance: A scoping review. BMC Medical Informatics and Decision Making. 2020;20(1):1–10. doi: 10.1186/s12911-020-01156-4 32782025 PMC7422501

[2] Dergaa I, Varma A, Tabben M, Malik RA, Sheik S, Vedasalam S, et al. Organising football matches with spectators during the COVID-19 pandemic: What can we learn from the Amir Cup Football Final of Qatar 2020? A call for action. Biology of Sport. 2021;38(4):677–681. doi: 10.5114/biolsport.2021.103568 34937978 PMC8670791

[3] Mao L, Peng Z, Liu H, Gómez MA. Identifying keys to win in the Chinese professional soccer league. International Journal of Performance Analysis in Sport. 2016;16(3):935–947. doi: 10.1080/24748668.2016.11868940

[4] Razali N, Mustapha A, Utama S, Din R. A review on football match outcome prediction using bayesian networks. In: Journal of Physics: Conference Series. vol. 1020. IOP Publishing; 2018. p. 012004.

[5] Almulla J, Alam T. Machine learning models reveal key performance metrics of football players to win matches in qatar stars league. IEEE Access. 2020;8:213695–213705. doi: 10.1109/ACCESS.2020.3038601

[6] Geurkink Y, Boone J, Verstockt S, Bourgois JG. Machine learning-based identification of the strongest predictive variables of winning and losing in Belgian professional soccer. Applied Sciences. 2021;11(5):2378. doi: 10.3390/app11052378

[7] Ahmad M, Sanawar S, Alfandi O, Qadri SF, Saeed IA, Khan S, et al. Facial expression recognition using lightweight deep learning modeling. Mathematical biosciences and engineering: MBE. 2023;20(5):8208–8225. doi: 10.3934/mbe.2023357 37161193

[8] Ahmad M, Qadri SF, Qadri S, Saeed IA, Zareen SS, Iqbal Z, et al. A lightweight convolutional neural network model for liver segmentation in medical diagnosis. Computational Intelligence and Neuroscience. 2022;2022. doi: 10.1155/2022/7954333 35755754 PMC9225858

[9] Hirra I, Ahmad M, Hussain A, Ashraf MU, Saeed IA, Qadri SF, et al. Breast cancer classification from histopathological images using patch-based deep learning modeling. IEEE Access. 2021;9:24273–24287. doi: 10.1109/ACCESS.2021.3056516

[10] Ozkan IA. A novel basketball result prediction model using a concurrent neuro-fuzzy system. Applied Artificial Intelligence. 2020;34(13):1038–1054. doi: 10.1080/08839514.2020.1804229

[11] Sikka D, D R. Basketball Win Percentage Prediction using Ensemble-based Machine Learning. In: 2022 6th International Conference on Electronics, Communication and Aerospace Technology; 2022. p. 885–890.

[12] C AT, S SH, H K KK, Aradhya R, S PB. Machine Learning Based Prediction OfThe Best Suitable Playing Positions of The Players In The Game of Basketball. In: 2021 IEEE Mysore Sub Section International Conference (MysuruCon); 2021. p. 232–237.

[13] Tümer AE, Koçer S. Prediction of team league’s rankings in volleyball by artificial neural network method. International Journal of Performance Analysis in Sport. 2017;17(3):202–211. doi: 10.1080/24748668.2017.1331570

[14] Gabrio A. Bayesian hierarchical models for the prediction of volleyball results. Journal of Applied Statistics. 2021;48(2):301–321. doi: 10.1080/02664763.2020.1723506 35707690 PMC9042147

[15] Huang ML, Li YZ. Use of machine learning and deep learning to predict the outcomes of major league baseball matches. Applied Sciences. 2021;11(10):4499. doi: 10.3390/app11104499

[16] Li SF, Huang ML, Li YZ. Exploring and Selecting Features to Predict the Next Outcomes of MLB Games. Entropy. 2022;24(2):288. doi: 10.3390/e24020288 35205582 PMC8871522

[17] O’Donoghue P, Ball D, Eustace J, McFarlan B, Nisotaki M. Predictive models of the 2015 Rugby World Cup: accuracy and application. International Journal of Computer Science in Sport. 2016;15(1):37–58. doi: 10.1515/ijcss-2016-0003

[18] McCabe A, Trevathan J. Artificial intelligence in sports prediction. In: Fifth International Conference on Information Technology: New Generations (itng 2008). IEEE; 2008. p. 1194–1197.

[19] Arabzad SM, Tayebi Araghi ME, Sadi-Nezhad S, Ghofrani N. Football match results prediction using artificial neural networks; the case of Iran Pro League. Journal of Applied Research on Industrial Engineering. 2014;1(3):159–179.

[20] Prasetio D, et al. Predicting football match results with logistic regression. In: 2016 International Conference On Advanced Informatics: Concepts, Theory And Application (ICAICTA). IEEE; 2016. p. 1–5.

[21] Arntzen H, Hvattum LM. Predicting match outcomes in association football using team ratings and player ratings. Statistical Modelling. 2021;21(5):449–470. doi: 10.1177/1471082X20929881

[22] Malamatinos MC, Vrochidou E, Papakostas GA. On Predicting Soccer Outcomes in the Greek League Using Machine Learning. Computers. 2022;11(9):133. doi: 10.3390/computers11090133

[23] Constantinou AC. Dolores: a model that predicts football match outcomes from all over the world. Machine Learning. 2019;108(1):49–75. doi: 10.1007/s10994-018-5703-7

[24] Guan S, Wang X. Optimization analysis of football match prediction model based on neural network. Neural Computing and Applications. 2022; p. 1–17.

[25] Pugsee P, Pattawong P. Football match result prediction using the random forest classifier. In: Proceedings of the 2nd International Conference on Big Data Technologies; 2019. p. 154–158.

[26] Carloni L, De Angelis A, Sansonetti G, Micarelli A. A machine learning approach to football match result prediction. In: HCI International 2021-Posters: 23rd HCI International Conference, HCII 2021, Virtual Event, July 24–29, 2021, Proceedings, Part II 23. Springer; 2021. p. 473–480.

[27] Bilek G, Ulas E. Predicting match outcome according to the quality of opponent in the English premier league using situational variables and team performance indicators. International Journal of Performance Analysis in Sport. 2019;19(6):930–941. doi: 10.1080/24748668.2019.1684773

[28] Elmiligi H, Saad S. Predicting the Outcome of Soccer Matches Using Machine Learning and Statistical Analysis. In: 2022 IEEE 12th Annual Computing and Communication Workshop and Conference (CCWC). IEEE; 2022. p. 1–8.

[29] Rico-González M, Pino-Ortega J, Méndez A, Clemente F, Baca A. Machine learning application in soccer: a systematic review. Biology of sport. 2023;40(1):249–263. doi: 10.5114/biolsport.2023.112970 36636183 PMC9806754

[30] Nyquist R, Pettersson D. Football match prediction using deep learning; 2017.

[31] Danisik N, Lacko P, Farkas M. Football match prediction using players attributes. In: 2018 World Symposium on Digital Intelligence for Systems and Machines (DISA). IEEE; 2018. p. 201–206.

[32] Rahman M, et al. A deep learning framework for football match prediction. SN Applied Sciences. 2020;2(2):1–12. doi: 10.1007/s42452-019-1821-5

[33] Tiwari E, Sardar P, Jain S. Football match result prediction using neural networks and deep learning. In: 2020 8th International Conference on Reliability, Infocom Technologies and Optimization (Trends and Future Directions)(ICRITO). IEEE; 2020. p. 229–231.

[34] Martins RG, Martins AS, Neves LA, Lima LV, Flores EL, do Nascimento MZ. Exploring polynomial classifier to predict match results in football championships. Expert Systems with Applications. 2017;83:79–93. doi: 10.1016/j.eswa.2017.04.040

[35] Rudrapal D, Boro S, Srivastava J, Singh S. A deep learning approach to predict football match result. In: Computational Intelligence in Data Mining. Springer; 2020. p. 93–99.

[36] SportVU;. Available from: https://www.statsperform.com/team-performance/football-performance/optical-tracking/.

[37] Kingma DP, Ba J. Adam: A method for stochastic optimization. arXiv preprint arXiv:14126980. 2014;.

[38] Smith LN, Topin N. Super-convergence: Very fast training of neural networks using large learning rates. In: Artificial intelligence and machine learning for multi-domain operations applications. vol. 11006. SPIE; 2019. p. 369–386.

[39] Cawley GC, Talbot NL. On over-fitting in model selection and subsequent selection bias in performance evaluation. The Journal of Machine Learning Research. 2010;11:2079–2107.

[40] Chen T, Guestrin C. Xgboost: A scalable tree boosting system. In: Proceedings of the 22nd acm sigkdd international conference on knowledge discovery and data mining; 2016. p. 785–794.

[41] Dellal A, Chamari K, Wong dP, Ahmaidi S, Keller D, Barros R, et al. Comparison of physical and technical performance in European soccer match-play: FA Premier League and La Liga. European journal of sport science. 2011;11(1):51–59. doi: 10.1080/17461391.2010.481334

